# Analgesia considerations in orthopaedic surgery: the role of magnesium sulfate infusions

**DOI:** 10.1051/sicotj/2025030

**Published:** 2025-06-06

**Authors:** Thomas J. Papadimos, Scott M. Pappada, Jacob Alexander, Pavlos Altsitzioglou, Theodosis Saranteas, Sebastien Lustig, Andreas F. Mavrogenis

**Affiliations:** 1 Department of Anesthesiology, Surgery, and Medical Microbiology and Immunology, University of Toledo College of Medicine and Life Sciences Toledo OH 43606-3390 USA; 2 Department of Anesthesiology, University of Toledo College of Medicine and Life Sciences Toledo OH 43606-3390 USA; 3 Department of Orthopaedic Surgery, University of Toledo College of Medicine and Life Sciences Toledo OH 43606-3390 USA; 4 First Department of Orthopaedics, National and Kapodistrian University of Athens, School of Medicine Athens 11527 Greece; 5 Second Department of Anesthesiology, National and Kapodistrian University of Athens, School of Medicine Athens 11527 Greece; 6 Orthopaedics Surgery and Sports Medicine Department, FIFA Medical Center of Excellence, Croix-Rousse Hospital, Hospices Civils de Lyon, Lyon University Hospital 103 Grande Rue de la Croix-Rousse 69004 Lyon France; 7 Univ Lyon, Claude Bernard Lyon 1 University, IFSTTAR, LBMC UMR_T9406 25 Avenue François Mitterand 69622 Lyon France

**Keywords:** Magnesium sulfate, Pain, Orthopaedic surgery

## Abstract

Orthopaedic surgical operations are associated with significant post-operative pain, often managed with opioids, which carry risks of adverse effects and dependency. Magnesium sulfate, a NMDA receptor antagonist with analgesic and muscle relaxant properties, has emerged as a potential adjunct to improve pain control and reduce opioid consumption in orthopaedic procedures. Current evidence supports magnesium sulfate as a valuable adjunct in orthopaedic pain management, particularly in reducing opioid consumption and enhancing muscle relaxation. However, heterogeneity in study design, administration protocols, and patient populations warrants cautious interpretation. Monitoring for side effects such as hypotension and respiratory depression remains essential. Further high-quality, standardized trials are needed to optimize dosing strategies and confirm long-term benefits.

## Introduction

**Figure 1:**
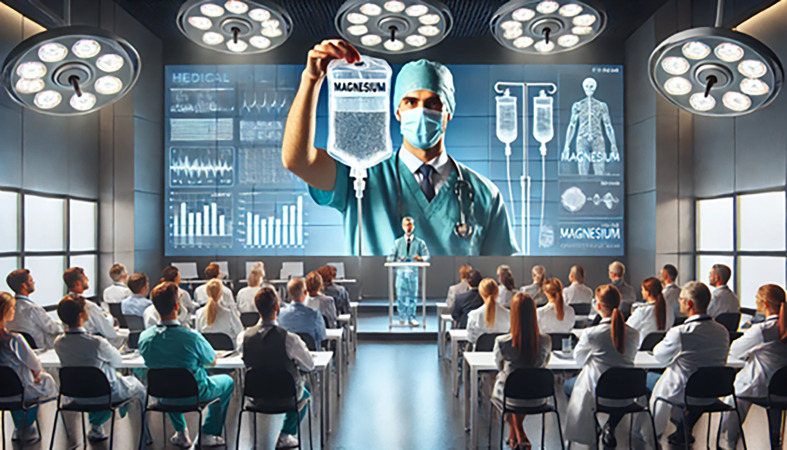
Controlling pain and minimizing opioids is essential in orthopaedic surgery.

There are one billion musculoskeletal injuries worldwide each year [1]. The European Union had 2.7 million fragility fractures in 2017 for €37.7 billion annually, with a 27% increase expected by 2030 [2]. The US has over seven million orthopaedic injuries each year, usually caused by motor vehicle crashes, sports injuries, and occupational accidents, and this results in 3.6 million hospital visits, 800,000 inpatient stays, and 600,000 emergency surgeries annually [3]. Furthermore, with increasing life expectancies and aging populations, especially in Europe, North America, and Japan, the orthopaedic community will face multiple clinical challenges [1]. Effective control of post-operative pain, while minimizing opioid use, is a key goal of modern orthopaedic care (Fig. 1).

Intravenous (IV) magnesium infusions can be incorporated into multimodal analgesic regimens to enhance pain control and reduce reliance on opioids. Pain management following orthopaedic surgery is a critical aspect of patient care, as inadequate control of post-operative pain can impair recovery, increase the risk of complications, prolong hospital length of stay, and cost institutions thousands of dollars in excess care and expenses [4–12].

The cost of the opioid epidemic in the US has taken its toll on patients, their families, and communities. This epidemic is costing the US more than $78 billion a year [13, 14]. While the same crisis is not manifest in Europe up to now, the synthetic opioid crisis may be reaching its doorstep shortly [15]. This crisis, in addition to the recent shortage of medications for surgical theaters, has required anesthesiologists to search for non-opiate solutions for their institutions and practices [16].

Conventional analgesic strategies, including opioids and nonsteroidal anti-inflammatory drugs (NSAIDs), are commonly used but may be associated with significant side effects, such as sedation, respiratory depression, gastrointestinal complications, and an increased risk of dependency [17–20]. As a result, there is growing interest in adjunctive therapies that can enhance analgesic efficacy while minimizing adverse effects [21, 22]. Magnesium has been studied extensively and used in multiple post-operative settings [23–30], as well as in chronic, neuropathic, and migraine pain. Its application in pain control through its potential ability to limit the use of perioperative opioids in orthopaedic surgery should be seriously considered.

## Magnesium sulfate properties of pain

Magnesium sulfate, administered via intravenous infusion, has emerged as a promising adjuvant in the management of post-operative pain. Intravenous magnesium sulfate has demonstrated effectiveness in reducing postoperative pain intensity, potentially decreasing the need for opioids, improving patient comfort, and reducing opioid side effects (e.g., nausea, constipation). Intravenous magnesium may also provide direct analgesia and reduce opioid-related complications through an opioid sparing effect [31–33]. Such infusions may also *reduce the inflammatory response* [34–36], which may delay recovery and contribute to further pain. Additionally, magnesium acts as a *muscle relaxant* [30, 37, 38], which may enhance surgical conditions, particularly in procedures requiring muscle relaxation such as hip arthroplasties, and may assist orthopaedic surgeons by providing significant muscle relaxation, allowing more effective intraoperative manipulation and reduction of spasms. Also, patients with chronic pain from various conditions may benefit from magnesium’s neuromodulatory effect [33, 39], particularly in conditions such as fibromyalgia and chronic postoperative pain syndromes.

More specifically, magnesium plays a vital role in modulating pain through its actions as a non-competitive antagonist of the N-methyl-D-aspartate (NMDA) receptor by blocking NMDA receptors in a voltage-dependent manner [40]. This NMDA antagonism is thought to reduce central sensitization, a key mechanism in acute and chronic pain. Magnesium also inhibits voltage-gated calcium channels, thereby decreasing neurotransmitter release (e.g., glutamate) and dampening nociceptive signaling [41]. This competition with calcium at the neuromuscular junction facilitates muscle relaxation and alleviates muscle tension or spasm. Furthermore, there is a downregulation of pro-inflammatory cytokines by magnesium (Tumor Necrosis Factor [TNF-α], Interleukin-6 [IL-6], and Interleukin beta 1 [IL-β1]), potentially minimizing postoperative pain and swelling [35, 42–46].

Magnesium infusions can be given before anesthetic induction, after anesthetic induction, intraoperatively, or postoperatively, depending on the protocol used, to mitigate intraoperative and postoperative pain [47] and may reduce the risk of developing chronic pain after surgery [12, 28, 48]. While magnesium infusions are generally safe [10], they require monitoring for side effects such as hypotension, bradycardia, and respiratory depression, especially in those with organ dysfunction [49].

**Figure 2:**
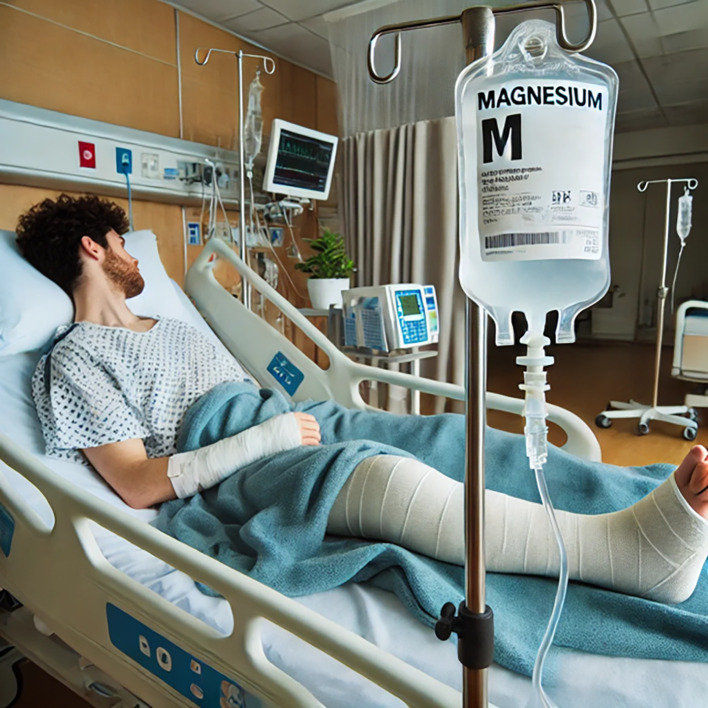
Intravenous magnesium sulfate infusion in the operating room, intraoperatively, and postoperatively, can decrease the use of opioids.

Additionally, intraarticular and intrathecal injections of magnesium sulfate have also been used successfully as an adjunct to local anesthetics and other analgesics in lower and upper extremity surgeries, including hip arthroplasties, to reduce pain and narcotic use [7, 50–52]. Magnesium sulfate infusions can be an invaluable adjunct for pain control in orthopaedic patients, both on the wards and in the intensive care unit (Fig. 2). While intravenous magnesium sulfate infusion protocols vary, specific dosing strategies (Table 1) and related side effects (Table 2) for various orthopaedic procedures have been reported.

**Table 1:** Dosing of magnesium sulfate in orthopaedic surgery.

Type of surgery	Dose (bolus, mg/kg, over 15 min)	Time before general anesthesia induction (minutes)	Time after spinal anesthesia induction (minutes)	Infusion rate (mg/kg/h, until end of surgery)
Upper extremity	30	15	Not applicable	10
Lumbar discectomy	50	10	Not applicable	10
Total knee arthroplasty*	50	Not applicable	<5	15
Hip arthroplasty*	50	Not applicable	<5	15

*Assumption that spinal anesthetic will be administered.

**Table 2:** Side effects of magnesium sulfate. (DTRs: deep tendon reflexes; ECG: electrocardiogram; PTH: parathyroid hormone).

Serum level	Symptoms	Signs	ECG concerns	Other
4–6 mEq/L	Nausea, vomiting, headache	Decreased DTRs and hypotension due to vasodilation	–	Associated with increased potassium and decreased calcium levels
6–10 mEq/L	Nausea, vomiting, lethargy and headache	Absent DTRs, bradycardia, and hypotension	Increased QRS duration and propagation of PR and QT intervals	Increased PTH release leading to decreased calcium levels
>10 mEq/L		Coma, paralysis, respiratory failure, and cardiac arrest	Complete heart block	

## Magnesium sulfate efficacy in orthopaedic surgery

Multiple systematic reviews and meta-analyses on the value of magnesium as a pain adjuvant in orthopaedic surgery have been published [5, 6, 9, 30, 53, 54] (Table 3). Gormley et al. addressed the reduction of opioid use after orthopaedic surgery [53]. These authors’ impetus was to limit opioid use during the current, ongoing, and rapidly increasing opioid epidemic in North America [13, 14]. They reviewed 141 studies of 20,963 patients; 113 were randomized controlled trials (RCTs). While all the studies used multiple interventions to reduce opioid use after orthopaedic surgery, only 4/141 (2.8%) addressed magnesium sulfate use. The authors emphasized the need for a rigorous and consistent application of methodology to RCTs in orthopaedic pain control to enable a thoughtful and helpful evidence-based approach to pharmacologic and non-pharmacologic interventions in clinical practice. This was especially true as it applies to magnesium sulfate infusions.

**Table 3:** Summary of the most important published related studies on magnesium sulfate intravenous infusions in orthopaedic surgery. (IT: intrathecal; IV: intravenous).

Study	Studies/patients (*n*)	Surgical focus	Key findings	Conclusions/notes
Gormley et al. [53]	141 studies/20,963 patients (113 RCTs)	Orthopaedic surgery	Only 4 studies (2.8%) involved magnesium sulfate; overall review on opioid-sparing strategies	Highlighted need for better RCT methodology and more evidence on magnesium use
Peng et al. [9]	11 RCTs/535 patients	Perioperative IV Magnesium in Orthopaedic Surgery	Six studies showed pain reduction; 5 did not. Reduced nausea, vomiting, and shivering	Mixed analgesic results; beneficial for perioperative symptom reduction
Sbitan et al. [6]	9 RCTs	IT vs. IV Magnesium in Orthopaedic Surgery	Only 3 trials directly compared IT vs. IV; both routes showed efficacy	Mode of administration (IT vs. IV) remains an unsolved issue
Campos et al. [30]	8 RCTs/541 patients	Spinal surgery	↓ Pain, ↓ opioid and muscle relaxant use; ↓ MAP vs. steroids; enhanced vecuronium effect	Magnesium is safe, effective, with synergistic benefits; dosing protocols vary
Yue et al. [54]	14 trials/781 patients	Spinal surgery	↓ Morphine use at 24 h	IV magnesium is effective in reducing postoperative opioid needs
Azimi et al. [5]	8 RCTs/536 patients	Total knee arthroplasty	↓ Opioid use in first 24 h; low-moderate evidence for analgesic efficacy	Heterogeneity in dosing/methods; more research needed

Peng et al. screened 2350 articles and found 11 RCTs (*N* = 535 patients). Their review also addressed perioperative IV administration of magnesium sulfate in orthopaedic surgery [9]. Their findings in support of magnesium yielded mixed results. They reported that six of the trials demonstrated a reduction in pain intensity, while five studies did not. However, they did report a reduction of nausea, vomiting, and shivering with the use of magnesium sulfate. Sbitan et al. performed a narrative review to compare the intrathecal (IT) or IV administration of magnesium sulfate as a pain adjuvant in orthopaedic procedures [6]. They identified nine RCTs that addressed IT and IV administration of magnesium sulfate, but only three trials directly compared the IT vs. IV approach. Their conclusion in this regard, for both approaches, was that there is evidence for the efficacy of magnesium. However, the choice of the mode of administration remained a critical question.

Campos et al. published a recent systematic review on the safety and efficacy of magnesium in spinal surgery [30]. They reported that the neuroprotective and anti-inflammatory effects of magnesium, compared to steroids, caused a decrease in pain. In doing so, magnesium also significantly decreased opioid consumption. Hemodynamically, when compared to steroids, magnesium demonstrated a lower mean arterial pressure (which may be a surgical advantage). Furthermore, magnesium also lowered the consumption of muscle relaxants and enhanced the effect of vecuronium. This analysis also confirmed that magnesium can be used in combination with other medications or strategies to elicit synergistic effects. Finally, the authors conclude that there is no agreement in the scientific community on whether to use a bolus, bolus plus infusion, or infusion only. Loading doses, maintenance dosing, and protocols varied. Most loading doses were initiated intravenously just prior to anesthetic induction, and ranged from 30 to 50 mg/kg in saline, and infused over 10–30 min; maintenance infusions were administered continuously throughout the surgery at a rate of 10–20 mg/kg/h with few side effects.

Yue et al. performed a systematic review and meta-analysis of fourteen trials (781 patients) that reported the use of IV magnesium infusions during spinal surgery [54]. They determined that magnesium infusions reduced morphine consumption at 24 h compared to controls [55–60]. The systematic review and meta-analysis by Azimi et al. explored RCTs and magnesium use in total knee arthroplasties (TKA) [5]. They evaluated eight RCTs regarding pain management and analgesic use in 536 patients. They also found that within the first 24 h postoperatively, there was a significant decrease in opioid consumption, but low to moderate evidence of the use of magnesium intraoperatively for postoperative pain control in TKAs. These authors complained of the heterogeneity of studies, methods, dosing regimens, and routes of administration. They advocated for continued research to assist the surgical and critical care communities.

## Conclusion

Despite some heterogeneity in study design, patient population, and dosing protocols, findings remain consistent. There is considerable evidence that magnesium infusions are effective in orthopaedic surgery, consistently demonstrating a reduction of postoperative opioid consumption and an improvement in pain scores when used in the first 24 h postoperatively. While it would be helpful to have further clinical studies with uniform methodological approaches (RCTs, surgery type, route of administration, dosing, etc.), and if possible, correlated with immunological (cytokine) findings, magnesium is becoming front and center for pain control in orthopaedic surgery. Multi-center trials examining the use of magnesium sulfate administration for pain control and reduced requests for analgesic medications in the perioperative period are worth pursuing. We encourage our colleagues in the surgical and anesthesiology communities to strongly consider adding magnesium sulfate infusions to their clinical pain armamentarium.

## Data Availability

This article has no associated data generated.
